# Genetic analysis of products of conception. Should we abandon classic karyotyping methodology?

**DOI:** 10.31744/einstein_journal/2021AO5945

**Published:** 2021-06-10

**Authors:** Denise Maria Christofolini, Leticia Busachero Bevilacqua, Fernanda Abani Mafra, Leslie Domenici Kulikowski, Bianca Bianco, Caio Parente Barbosa

**Affiliations:** 1 Centro Universitário FMABC Santo AndréSP Brazil Centro Universitário FMABC, Santo André, SP, Brazil.

**Keywords:** Karyotype, Abortion, spontaneous, Pregnancy, Aneuploidy, Chromosome deletion, Chromosome duplication

## Abstract

**Objective:**

To compare the results obtained by the classic and molecular methodology in the analysis of products of conception, the advantages and disadvantages of each method.

**Methods:**

Retrospective non-randomized analysis of results obtained from product of conception samples submitted to genetic evaluation, from 2012 to 2017. The evaluations were performed using cytogenetics and/or chromosomal microarray analysis or arrays.

**Results:**

Forty samples were analyzed using classic cytogenetics, of which 10% showed no cell growth, 50% had normal results and 40% had abnormalities. Of the 41 cases sent for array analysis it was not possible to obtain results in 7.3%, 39.5% were normal and 60.5% had abnormalities. There was no statistical difference among the results (p=0.89). Most abnormal results were seen till 9 weeks’ gestation. The later abnormal miscarriage was seen at 28 weeks’ gestation, with karyotype 46,XX,del(15)(q26.2-qter). The results are corroborated by the international literature.

**Conclusion:**

Classic cytogenetics and array techniques showed comparable results on the type of alteration observed. Array analysis is preferable to cell culture in delayed abortions, while cytogenetics is more able to show polyploidies. Both have the same growth failure rates when product of conception tissue is not properly collected.

## INTRODUCTION

Spontaneous abortion or miscarriage is one of the most common complications of pregnancy, being responsible for the significant emotional stress of the couple with reproductive desire. Some studies have demonstrated that between 10% and 15% of the clinically recognized gestations result in miscarriage during the first trimester of pregnancy.^(^^1^^,^^2^^)^

There are several causes related to abortion, such as maternal age, endocrine imbalances, autoimmune factors, infectious diseases, implantation or uterine abnormalities, and chromosomal abnormalities present in one of the parents or in the embryo.^(^^3^^)^

Classic cytogenetic analysis has shown that the most common causes of abortion in the first trimester are chromosomal abnormalities, seen in 50% of products of conception (POC). Most of these abnormalities are numerical (95%) – in that, 64% trisomies, 10% monosomy for the chromosome X, and the approximately remaining 15% polyploidies, especially triploidies.^(^^2^^,^^4^^)^ In addition to complete aneuploidies, partial monosomy and trisomy are also observed, half of them coming from a parent with balanced alterations, such as inversions and translocations.^(^^5^^,^^6^^)^

Cytogenetic analysis of the POC can clarify the cause of abortion and the risk of recurrence for the couple. However, this analysis depends on the successful cell culture of fetal tissue, and on the preparation of metaphasic cells, which depend on a well-established cytogenetic methodology in the routine laboratory. Besides, it takes between 2 to 6 weeks to obtain the result. However, regardless of the laboratory standardization, the literature shows a cell growth failure varying from 10 to 40% for POC, resulting in misdiagnosis.^(^^7^^)^

A factor that interferes with the success of POC cell culture is the period of time between the moment when the gestational loss occurred and the collection of the material. If there is a long time between the two events, the cells present in this tissue may no longer divide, preventing cytogenetic analysis. At this point, it is possible that cells derived from maternal (deciduous) tissue growth instead of fetal tissue, resulting in an erroneously normal karyotype.^(^^8^^)^ In addition, the method of collecting retained POC can also prevent cell culture, if there is no collection of chorionic villi or if the material is contaminated by fungi and bacteria from the female cervical flora.

With the advent of DNA-based analysis methods, such as chromosomal microarray analysis (CMA) and new generation sequencing (NGS), there is an alternative to cell culture. These techniques, however, are more expensive and not always available in developing countries, since their performance depends on sophisticated equipments. They must also be validated as to the possibility of execution and advantages over the existing techniques.

## OBJECTIVE

To compare the results obtained after classic cytogenetic and chromosomal microarray analysis of products of conception, observe the frequency of chromosomal alterations, and correlate with the ages and period of pregnancy loss, point out limitations of each technique.

## METHODS

This is a non-randomized retrospective descriptive study. A total of 81 fetal tissue samples were obtained from women who underwent spontaneous abortion from January 2012 to December 2017. Product of conception samples were sent to the genetics laboratory to clarify the cause of the abortion using classic cytogenetics or CMA. The choice of the test to be performed was not randomized but clinically indicated. The samples were sent to the laboratory in a sterile flask containing culture medium (classic cytogenetics) or preservative solution CMA provided by the laboratory. The physicians received an orientation brochure on the material that should be placed in the flasks for analysis.

The 40 samples sent for classic cytogenetics analysis were placed on a Petri dish containing a drop of cell culture medium. The chorionic villi were separated from the maternal decidua using a magnifying glass and microdissected with the aid of trypsin and collagenase. The sample was divided into four parts that were placed in four T25 flasks containing two different culture media (Gibco AmnioMAX and Chang Medium, Irvine Scientific) kept in an incubator at 37°C and 5% carbon dioxide atmosphere. After 80% of cells reaching confluence, colchicine was added to the cell culture medium, and the flasks were kept in the incubator during four hours. The cells were trypsinized, removed from the flask, and cell preparation was performed, using hypotonic solution and sequential washes with methanol:acetic acid (3:1). The cell preparation was spread over microscopic slides that were aged and stained for G banding. For each sample, 20 metaphases were selected for inspection in the largest magnification (1,000x), which were photographed and analyzed using the Ikaros software (Metasystems) for the presence of numerical and structural changes through the realization of a karyotype.

The 41 samples sent for analysis by CMA were sent to a reference laboratory, where they were processed for DNA extraction and single-nucleotide polymorphism (SNP) array. All CMA cases were evaluated using the CytoSNP-12 chip (Illumina, California, United States). Deviations from normality involving more than 200kb for gains, more than 50kb for losses, and more than 10kb for loss of heterozygosity were considered, and also gene content (specifically, dosage-sensitive genes with explicit disease association), overlap with a cytogenetically relevant deletion/duplication critical region. Along with the POC sample, a 5mL sample of maternal blood was also collected in a tube containing EDTA, to exclude maternal contamination and determine the parental origin of the chromosomal abnormality, if present.

Six cases referred for classic cytogenetics and seven cases for CMA, whose material sent was abundant, had part of the villus frozen in a 10% dimethyl sulfoxide solution. This samples were reevaluated by the array methodology in another research laboratory (Medical Investigation Laboratory, Department of Pathology, *Faculdade de Medicina* of *Universidade de São Paulo* – USP), to confirm previous results, evaluate reproducibility and viability of frozen samples.

### Statistical analysis

Statistical analysis was carried out using SPSS for Windows 11.0 (Inc., Chicago, IL, United States). The χ^2^ test was used to compare aneuploidy frequencies among the groups. A p-value<0.05 was considered statistically significant.

### Ethics

The research was approved by the *Faculdade de Medicina do ABC, Centro Universitário FMABC* Research Ethics Committee (CAAE: 64418417.5.0000.0082) protocol 1,976,135 and all patients signed a research consent.

## RESULTS

Forty cases were analyzed by classic cytogenetics, in long-term culture. In four cases (10%), it was not possible to obtain results due to cell culture failure. Of the 36 cases with cell growth, in which the karyotype was performed, 20 (55.5%) had a normal result (46,XX or 46,XY) and 16 had an abnormal result (44.5%). In one case, 46,XX and 46,XY cells were observed, possibly due to contamination with maternal cells. This case was considered as a normal male.

In three cases (7.32%) out of 41 analyzed by CMA, it was not possible to obtain results due to insufficient DNA or the exclusive presence of maternal cells. Of the 38 samples evaluated, 15 had a normal result (46,XX or 46,XY) (39.5%) and 23 had an abnormal result (60.5%), as shown in table 1.

**Table 1:** Comparative description of classic cytogenetics and chromosomal microarray analysis results

Results	Cytogenetics n (%)	CMA n (%)	χ^2^ (2 d.f.)	p value
Monosomy	4 (10.0)	2 (4.9)	2.102	0.35
Trisomy	6 (15.0)	18 (43.9)		
Structural rearrangement	2 (5.0)	1 (2.4)		
Poliploidy	4 (10.0)	2 (4.9)		
No result	4 (10.0)	3 (7.3)		
Normal	20 (50.0)	15 (36.6)		

CMA: chromosomal microarray analysis.

Considering the classic cytogenetics’ cases reassessed by CMA, four maintained the previous results, one case of previous failure to grow demonstrated a 46,XY result, and one case with cytogenetic results was not amplified in the array. Considering CMA results, six maintained the previous results and a triploidy resulted in amplification failure.

The most frequent chromosomal aberration found was trisomy, involving chromosomes 22 and 16 (11.1% and 4.9% respectively). We observed no abnormalities involving larger chromosomes (Groups A and B). Detailed frequencies of chromosome aberrations are available in the table 2. The structural changes observed were all deletions: 46,XX,del(6)(p23), 46,XX,del(12)(p13-pter) and 46,XX,del(15)(q26.2-qter)(pat). In the statistical comparison, no significant difference was found between the CMA and cytogenetic results, grouped into categories or individually.

**Table 2:** Distribution of the results obtained by classic cytogenetics and chromosomal microarray analysis of products of conception

Results		Classic cytogenetics n (%)	CMA n (%)
Normal		20 (50.0)	15 (36.6)
Inconclusive		4 (10.0)	3 (7.3)
Trisomies			
	Trisomy 9	0	2 (4.9)
	Trisomy 13	0	1 (2.4)
	Trisomy 14	0	1 (2.4)
	Trisomy 15*	2 (5.0)	1 (2.4)
	Trisomy 16	1 (2.5)	3 (7.3)
	Trisomy 17	1 (2.5)	0
	Trisomy 18	0	1 (2.4)
	Trisomy 21	0	1 (2.4)
	Trisomy 22	2 (5.0)	7 (17.1)
	Trisomy 9 and monosomy X	0	1 (2.4)
Monosomies			
	Monosomy X*	3 (7.5)	2 (4.8)
	Monosomy 21	1 (2.5)	0
Polyploidy*		4 (10.0)	2 (4.9)
Structural aberration		2 (5.0)	1 (2.4)
Total		40	41

* Universal or in a mosaic presentation.

CMA: chromosomal microarray analysis.

Maternal age at the time of pregnancy loss was also assessed. Table 3 shows the correlation between maternal age and period of pregnancy loss in weeks.

**Table 3:** Correlation between age, week of pregnancy loss and results found by classical cytogenetics and chromosomal microarray analysis analysis

Age/gestational loss		n (%)*	Results		
Up to 35 years old		32 (38.3)	Normal	Abnormal	Inconclusive
	Up to 5 weeks	0	0	0	0
	6-9 weeks	21 (25.9)	9	11	1
	10-12 weeks	5 (6.2)	4	1	0
	13-19 weeks	2 (2.4)	2	0	0
	More than 20 weeks	0	0	0	0
	No GA information	4 (4.9)	1	2	1
Age over 35 years		36 (44.4)	Normal	Abnormal	Inconclusive
	Up to 5 weeks	1 (1.2)	1	0	0
	6-9 weeks	27 (33.3)	7	17	3
	10-12 weeks	1 (1.2)	0	1	0
	13-19 weeks	1 (1.2)	0	1	0
	More than 20 weeks	1 (1.2)	0	0	1
	No GA information	5 (6.2)	3	2	0
No maternal age information		13 (16)	Normal	Abnormal	Inconclusive
	Up to 5 weeks	0	0	0	0
	6-9 weeks	5 (6.2)	3	1	1
	10-12 weeks	1 (1.2)	1	0	0
	13-19 weeks	0	0	0	0
	More than 20 weeks	2 (2.4)	1	1	0
	No GA information	5 (6.2)	4	1	0
Total		81 (100)	36	38	7

* Compared to total.

GA: gestational age.

**Figure 1:**
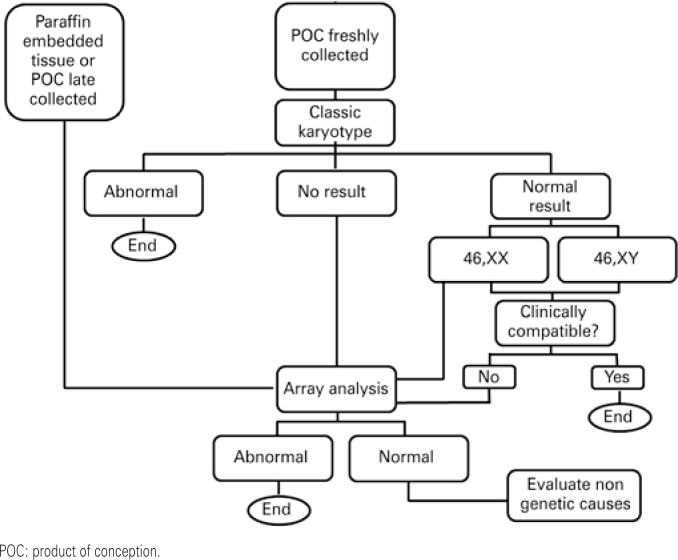
Decision-making chart considering the analysis of the product of conception

A chart was built to assist decision making by physicians and patients (Figure 1).

## DISCUSSION

Miscarriage is the major complication of pregnancy and a stressful event for the couple. To know the cause of miscarriage helps in the healing process for the couple, dismissing eventual guilt and allowing them to plan their reproductive future.

It is already known that from 45% to 70% of early miscarriages are due to chromosomal abnormalities in the embryo arising from maternal non-disjunction events.^(^^9^^–^^11^^)^ Due to his huge impact on fetal formation, the frequency of chromosome aberration decreases during pregnancy.^(^^12^^)^ In our sample, we observed that most of miscarriages occurred from 6 to 9 weeks’ gestation, concentrating 66.6% of gestational losses. Of these, 50.6% had chromosome aberrations.

The distribution of the various types of abnormalities considering aneuploidies, polyploidies and structural abnormalities was 72.5%, 15%, and 7.5% respectively. The most frequent aneuploidy type in the sample was the trisomy, observed in 70% of abnormal cases, 22% of them involving the chromosome 22. A meta-analysis considering 19,920 miscarriage cases showed trisomy accounts for 59% of abnormalities, monosomy X for 15%, and triploidy for 15%. The trisomies more often observed by them involved chromosomes 16 (32.1%), 7 and 22 (10.7% each).^(^^13^^)^ Moreover, a new research evaluated 1,920 POC and found 57.2% of abnormal results. The most frequent alteration involved chromosome 16 trisomy, accounting for 12% of abnormal cases, followed by chromosomes 21 and 22 trisomies.^(^^9^^)^

Among the structural aberrations, we observed a partial aneuploidy in a late abortion, which occurred during the 28^th^ week of gestation. This variant had a paternal origin and corresponded to a partial deletion of the long arm of chromosome 15 [46,XX,del(15)(q26.2-qter)]. The partial deletion of chromosome 6 [(46,XX,del(6)(p23)] resulted in an abortion in the 7^th^ week of gestation, and for the variant chromosome 12 aberration [46,XX,del(12)(p13-pter)] we do not have information on gestational age. The three changes were large and could have been observed by both methods. The frequency of structural changes is usually lower than other aberrations, as observed here (4%) and in the medical literature. A review considering 13 studies and 7,012 cases observed a 6% frequency of structural rearrangements in miscarriages.^(^^2^^)^

There was no difference in the success rate between the techniques used: in four cases of cytogenetics, there was cell growth failure, while three cases were inconclusive by CMA due to the insufficient DNA sample for analysis, or to exclusive presence of maternal cells. Failure was observed in 8.6% of analysis. The failure rate of cell culture for karyotype analysis verified in this study (10%) is slightly different from the rate of 12% to 21% described in the literature.^(^^2^^,^^14^^)^

Thirteen cases randomly selected were reevaluated by the CMA in another research laboratory. In one case of classic cytogenetics, previously inconclusive, a result could be achieved (46,XY). On the other hand, a case with cytogenetic result was not amplified in the array. Maybe the material was insufficient or not properly extracted. Concerning CMA samples, a triploidy resulted in amplification failure in the second study. The level of agreement observed was 66.6% and 85.7%, respectively.

Regarding gestational age, the results do not provide evidence of a significant association of age with sex chromosome monosomy or polyploidy, but do demonstrate an effect of age increase in the frequency of trisomies. Hassold et al.,^(^^15^^)^ and many other researchers who succeeded them had previously demonstrated the association of maternal age and trisomies. Franasiak et al.,^(^^16^^)^ in a large genetic preimplantation study of embryos, observed an increase of monosomies in patients aged under 26 years, and an increase of trisomies in women older than 34 years.

Here we observed among the trisomies diagnosed that 73.9% occurred in women aged over 35 years. The association of trisomies with maternal age is an increasing problem, since the average age at which women have their first child is currently 31 years, in Brazil, thus increasing chronologically in the South and Southeast regions of the country (with more access to education); and increasing proportionally to sociocultural level.^(^^17^^)^

In contrast, the results of abortion in women aged up to 35 years showed 48.3% had a normal karyotype, suggesting causes of abortion different from aneuploidies. Some studies suggest abortions without changes in the karyotype may be due to other causes (endocrine, immune, anatomical etc).^(^^18^^)^ Gene mutations and mosaicism were also implicated as causes of pregnancy losses.^(^^4^^)^

Still considering the maternal age, we observed 53.7% of abortions occurred in women aged over 35 years. Among the analyzed samples of these women, 66.7% had abnormal results. It is worth mentioning, however, that 17% of tests requested did not inform the mother’s age in the paperwork, and that the median (p.25; p.75) of the ages of the patients analyzed was 36 (32; 39) years.

Conventional karyotyping is still considered the gold standard of POC testing because it has important advantages over molecular techniques, including the detection of balanced tetraploidy (two copies from the mother and two copies from the father), and Robertsonian translocations.^(^^9^^)^ However, classic cytogenetics analysis is limited to a cell culture time that varies between 7 and 10 days.^(^^19^^)^ The slide analysis period should still be added to this time, providing the result to the couple between 15 days and one month, while in the analysis by molecular cytogenetics, the result can be obtained two days after sending the samples to analysis laboratory, depending on the routine of the examination. On the other hand, in Brazil, the cost of procedures is quite different, and CMA cost is almost four times the offered by classic cytogenetics investigation, and only few laboratories in the country perform this procedure. These factors should be considered when discussing with the couple the type of analysis they wish to proceed.

Considering the advantages of the techniques, CMA allows the identification of small deletions and duplications and uniparental disomy; but it is not able to detect balanced chromosomal rearrangements, or balanced poliploidy. Overall, polyploidy accounts for approximately 2% to 10% of all spontaneous abortions^(^^7^^)^ and is easily identifiable by classic cytogenetics. In our sample, it was present in 7.4% of cases. Chromosomal microarray analysis allows detecting contamination by maternal DNA (provided that the analysis of maternal blood is made), does not need cell culture, and offers the possibility of evaluating paraffin embedded material. The exclusion of maternal origin is of great importance in the analysis of the result, since it is known that between 29% and 58% of the results 46,XX, found by molecular analysis, are of maternal origin,^(^^2^^,^^7^^,^^8^^,^^20^^)^ which can lead to wrong conclusions about the analysis of the product of abortion. Another advantage of CMA by SNPs is the possibility of identifying the parental source of aneuploidy, information that has proven to be valuable in the couple’s reproductive counseling.^(^^21^^)^

Lathi et al., performed the simultaneous analysis of 30 samples by classic cytogenetics analysis and SNP array.^(^^21^^)^ They observed disagreement in four results (87%): the first referring to the analysis of a tetraploid sample, the second to determine a Robertsonian translocation derivative. In a third sample, classic cytogenetics demonstrated trisomy of chromosome 22, and the CMA analysis identified only cells of maternal origin. And in the fourth sample, the result 46,X,+22 was observed in the karyotype, while the SNP array reported 47,XX,+22.

In a meta-analysis of studies comparing CMA and classic cytogenetics, it was concluded that there was agreement between array and karyotyping in 86.0% of cases. The array detected 13% of chromosomal abnormalities in addition to the classic cytogenetics. On the other hand, cytogenetics detected additional abnormalities of 3% in relation to the array.^(^^22^^)^

Sahoo et al., evaluated 8,118 POC samples, fresh and formalin-fixed and paraffin-embedded tissues using SNP array (81.6%) and comparative genomic hybridization (CGH) array (18.4%). Analysis of 7,396 samples (91.1%) was successful, with 92.4% fresh tissue and 86.4% formalin-fixed and paraffin-embedded tissues. Clinically significant abnormalities were identified in 53.7% of samples, 94% of which were considered to be the cause of pregnancy loss.^(^^23^^)^

Shah et al., analyzed 60 samples simultaneously by cytogenetics, SNP array, and CGH array testing. They found a 33% overall discordance rate in results, due to maternal cell contamination, balanced chromosome rearrangements, polyploidy, and placental mosaicism (detected in 18% of all samples). Growth failure occurred in four samples sent to cytogenetics, of which three were chromosomally abnormal by molecular testing.^(^^24^^)^

Regardless of the method chosen, we know that without chromosomal evaluation, 80% of recurrent pregnancy losses remain with no etiological clarification. Of these, 62% would have chromosomal abnormalities, whereas the other 18% would remain unclear after classic karyotype.^(^^25^^)^ Additionally, Ouyang et al., demonstrated the chance of finding chromosomal changes in the POC depends on the gestational age, being more frequent in the embryonic stage than in the fetal stage. They were also more frequent in embryonic pregnancies than in non-embryonic pregnancies.^(^^26^^)^

Segawa et al.,^(^^27^^)^ performed an important cytogenetic study of 1,030 POC generated after embryo transfer in patients submitted to *in vitro* fertilization procedures. They observed that 80.6% of them were aneuploid, including 1% polyploid and 1.1% of mosaic karyotypes. Polyploidy and normal karyotypes were significantly more frequent in gestations with no fetal heartbeat. They also observed 2.4% of structural abnormalities, corroborating our spontaneous abortion results.

Considering gestational age of the POC evaluated in the present study, 89.4% happened during the first gestational trimester. Of the eight patients with gestational age equal to or higher than 12 weeks, only two (25%) presented genetic abnormalities (one detected by cytogenetics and the other detected by CMA) and one showed no cellular growth.

We recognize as limitations of our study that few samples were submitted to both tests. Although we cannot directly compare the results, we did not observe different frequency of aneuploidies in the groups, or results that would be observed by only one method. Even the structural rearrangements could have been detected by both methodologies. In addition, only classic cytogenetics indicated the presence of mosaicism, but current molecular techniques allow this detection, as long as they are in a proportion larger than 15%.

Thus, considering the data mentioned, we suggest that POC should be first evaluated by classic cytogenetics, considered the gold standard in the analysis of the POC, preserving (frozen or formalin-fixed and paraffin-embedded tissues), when possible, part of the genetic material for later molecular analysis in cases with 46,XX results, normal results with incompatible clinic, or cases with cell culture failure, mainly in women aged over 35 years.

## CONCLUSION

There was no difference in the results of chromosomal microarray analysis and classic cytogenetics concerning the frequency of chromosomal aberrations observed. Inconclusive results were found in similar proportion in both techniques. Abnormal results were more frequently observed up to 9 weeks of gestation and in women aged 35 years or more.
