# Supplementary material for: Genetic analysis of products of conception. Should we abandon classic karyotyping methodology?
Source: Einstein (Sao Paulo). 2021 Jun 10;19:eAO5945. doi: 10.31744/einstein_journal/2021AO5945 (PMC8225262; doi:10.31744/einstein_journal/2021AO5945)
Supplement: Translation

# Translation: Análise genética dos produtos de concepção. Devemos abandonar a metodologia de cariotipagem clássica?

**Authors:** Denise Maria Christofolini, Leticia Busachero Bevilacqua, Fernanda Abani Mafra, Leslie Domenici Kulikowski, Bianca Bianco, Caio Parente Barbosa

**Affiliations:** 1 Santo AndréSP Brasil Centro Universitário FMABC, Santo André, SP, Brasil.

**Keywords:** Cariótipo, Aborto espontâneo, Gravidez, Aneuploidia, Deleção cromossômica, Duplicação cromossômica

## Abstract

**Objetivo:**

Comparar os resultados obtidos pela metodologia clássica e molecular na análise de produtos de concepção, além das vantagens e desvantagens de cada método.

**Métodos:**

Análise retrospectiva não randomizada dos resultados obtidos a partir de amostras de produto de concepção submetidas à avaliação genética, de 2012 a 2017. As análises foram realizadas por citogenética clássica e/ou análise cromossômica de *microarray* ou *arrays*.

**Resultados:**

Quarenta amostras foram analisadas por citogenética, das quais 10% não apresentaram crescimento celular, 50% apresentaram resultados normais, e 40% apresentaram anormalidades. Dos 41 casos encaminhados para análise por *array*, não foi possível obter resultados em 7,3%, 39,5% eram normais, e 60,5% apresentavam alterações. Não houve diferença estatística entre os resultados (p=0,89). A maioria dos resultados anormais foi observada até a nona semana de gestação. Uma perda fetal mais tardia foi observada na 28ª semana de gestação, com cariótipo 46,XX,del(15)(q26.2-qter). Os números observados corroboraram a literatura mundial.

**Conclusão:**

As técnicas de citogenética clássica e análise por *array* mostraram resultados comparáveis no tipo de alteração observada. O *array* é preferível à cultura de células em abortos tardios, enquanto a citogenética é mais capaz de mostrar poliploidias. Ambos têm as mesmas taxas de falha de crescimento quando o tecido do produto de concepção não é coletado adequadamente.

## INTRODUÇÃO

O aborto espontâneo é uma das complicações mais comuns da gravidez, sendo responsável por significativo estresse emocional do casal com desejo reprodutivo. Alguns estudos demonstraram que entre 10% e 15% das gestações clinicamente reconhecidas resultam em aborto durante o primeiro trimestre da gravidez.^(^^1^^,^^2^^)^

São várias as causas relacionadas ao aborto, como idade materna, desequilíbrios endócrinos, fatores autoimunes, doenças infecciosas, anomalias de implantação ou uterinas e anomalias cromossômicas presentes em um dos membros do casal ou no embrião.^(^^3^^)^

A análise citogenética clássica mostrou que as causas mais comuns de aborto no primeiro trimestre são anomalias cromossômicas, vistas em 50% dos produtos de concepção (POC). A maioria dessas anormalidades é numérica (95%), sendo 64% de trissomias, 10% de monossomias do cromossomo X e os aproximadamente 15% restantes de poliploidias, especialmente triploidias.^(^^2^^,^^4^^)^ Além das aneuploidias completas, também são observadas trissomias e monossomias parciais, metade delas proveniente de um dos pais com alterações balanceadas, como inversões e translocações.^(^^5^^,^^6^^)^

A análise citogenética do POC pode esclarecer a causa do aborto e o risco de recorrência para o casal. No entanto, essa análise depende do sucesso da cultura de células de tecido fetal e da preparação de células metafásicas, que dependem de uma metodologia citogenética bem estabelecida em laboratório de rotina. Além disso, leva entre 2 e 6 semanas para se obter o resultado. No entanto, independente da padronização do laboratório, a literatura mostra que há um índice de falha no crescimento celular que varia de 10% a 40% para POC, resultando em diagnóstico incorreto.^(^^7^^)^

Um fator que interfere no sucesso da cultura de células do POC é o período de tempo decorrido entre o momento em que ocorreu a perda gestacional e a coleta do material. Se houver um tempo muito longo entre os dois eventos, as células presentes nesse tecido podem não se dividir mais, impedindo a análise citogenética. Nesse ponto, é possível que cresçam células derivadas de tecido materno (decidual) em vez de tecido fetal, resultando em um cariótipo erroneamente normal.^(^^8^^)^ Além disso, o método de coleta do POC retido também pode impedir a cultura de células, se não houver coleta de vilosidades coriônicas ou se o material estiver contaminado por fungos e bactérias da flora feminina do cérvix.

Com o advento de metodologias de análise baseadas em DNA, como a análise cromossômica por *microarrays* (CMA - *chromosomal microarray analysis*) e o sequenciamento de nova geração (NGS - *new generation sequencing*), surge uma alternativa ao cultivo celular. Essas técnicas, entretanto, são mais caras e nem sempre estão disponíveis nos países em desenvolvimento, pois dependem de equipamentos sofisticados para sua realização. Devem também ser validados quanto à possibilidade de execução e vantagens sobre as técnicas existentes.

## OBJETIVO

Comparar os resultados obtidos por análise citogenética clássica e análise cromossômica por *microarrays* dos produtos da concepção; observar a frequência das alterações cromossômicas e correlacioná-las com as idades e o período da perda gestacional e apontar as limitações de cada técnica.

## MÉTODOS

Este é um estudo descritivo retrospectivo não randomizado. Foram obtidas 81 amostras de tecido fetal de mulheres que tiveram aborto espontâneo de janeiro de 2012 a dezembro de 2017. As amostras de produtos da concepção foram enviadas ao laboratório de genética, para esclarecimento da causa do aborto por citogenética clássica ou CMA. A escolha do teste a ser realizado não foi randomizada, mas clinicamente indicada. As amostras foram enviadas para o laboratório em frasco estéril contendo meio de cultura (citogenética clássica) ou solução conservante (CMA) fornecida pelo laboratório. Os médicos receberam um folheto de orientação sobre o material que deveria ser depositado nos frascos para análise.

As 40 amostras enviadas para análise citogenética clássica foram colocadas em uma placa de Petri contendo uma gota de meio de cultura celular. As vilosidades coriônicas foram separadas da decídua materna por meio de lupa e microdissecadas com auxílio de tripsina e colagenase. A amostra foi dividida em quatro partes, que foram colocadas em quatro frascos T25, contendo dois meios de cultura diferentes (Gibco AmnioMAX e Chang Medium, Irvine Scientific) mantidos em incubadora a 37°C, em atmosfera de dióxido de carbono a 5%. Após 80% das células atingirem a confluência, foi adicionada colchicina ao meio de cultura celular, e os frascos foram mantidos na incubadora por 4 horas. As células foram tripsinizadas e retiradas do frasco, e foi realizada a preparação das células, utilizando solução hipotônica e lavagens sequenciais com metanol:ácido acético (3:1). O preparado de células foi espalhado sobre lâminas para microscopia, que foram envelhecidas e coradas para bandas G. Para cada amostra foram selecionadas 20 metáfases para inspeção no maior aumento (1.000x), as quais foram fotografadas e analisadas no software Ikaros (Metasystems) quanto à presença de alterações numéricas e estruturais por meio da realização de um cariótipo.

As 41 amostras enviadas para análise por CMA foram encaminhadas a um laboratório de referência, onde foram processadas para extração de DNA e *arrays* de polimorfismo de nucleotídeo único (SNP - *single-nucleotide polymorphism*). Todos os casos de CMA foram avaliados com o CytoSNP-12 Beadchip (Illumina, Califórnia, Estados Unidos). Foram considerados desvios da normalidade envolvendo mais de 200kb para ganhos, mais de 50kb para perdas e mais de 10kb para perda de heterozigosidade, além do conteúdo do gene (especificamente genes sensíveis à dosagem com associação de doença explícita), com sobreposição a uma região crítica e citogeneticamente relevante de deleção/duplicação. Junto com a amostra POC, uma amostra de 5mL de sangue materno também foi coletada em um tubo contendo EDTA, para excluir a contaminação materna e determinar a origem parental da anomalia cromossômica, se presente.

Seis casos encaminhados para citogenética clássica e sete casos para CMA, cujo material enviado foi abundante, tiveram parte da vilosidade congelada em solução de *dimetilsulfóxido* a 10%. Essas amostras foram reavaliadas pela metodologia de *array* em outro laboratório de pesquisa (Laboratório de Investigação Médica do Departamento de Patologia da Faculdade de Medicina da Universidade de São Paulo – USP), para confirmar os resultados anteriores e avaliar a reprodutibilidade e a viabilidade das amostras congeladas.

### Análise estatística

A análise estatística foi realizada usando SPSS para Windows 11.0 (Inc., Chicago, IL, Estados Unidos). O teste do χ^2^ foi usado para comparar as frequências de aneuploidia entre os grupos. Valor de p<0,05 foi considerado estatisticamente significativo.

### Ética

A pesquisa foi aprovada pelo Comitê de Ética em Pesquisa do Faculdade de Medicina do ABC, Centro Universitário FMABC (CAAE: 64418417.5.0000.0082), protocolo 1.976.135, e todos os pacientes assinaram o consentimento de pesquisa.

## RESULTADOS

Quarenta casos foram analisados por citogenética clássica, em cultura de longa duração. Em quatro casos (10%), não foi possível obter resultados por falha na cultura de células. Dos 36 casos com crescimento celular, nos quais foi realizado o cariótipo, 20 (55,5%) tiveram resultado normal (46,XX ou 46,XY) e 16 tiveram resultado anormal (44,5%). Em um caso, foram observadas células 46,XX e 46,XY, possivelmente devido à contaminação com células maternas. Esse caso foi considerado masculino normal.

Em três casos (7,32%) dos 41 analisados por CMA, não foi possível de se obterem resultados por DNA insuficiente ou por presença exclusiva de células maternas. Das 38 amostras avaliadas, 15 tiveram resultado normal (46,XX ou 46,XY) (39,5%) e 23 anormal (60,5%), conforme mostra a tabela 1.

**Tabela 1:** Descrição comparativa da citogenética clássica e resultados da análise cromossômica por *microarrays*

Resultados	Citogenética n (%)	CMA n (%)	χ^2^ (2 d.f.)	Valor de p
Monossomia	4 (10,0)	2 (4,9)	2,102	0,35
Trissomia	6 (15,0)	18 (43,9)		
Reorganização estrutural	2 (5,0)	1 (2,4)		
Poliploidia	4 (10,0)	2 (4,9)		
Sem resultado	4 (10,0)	3 (7,3)		
Normal	20 (50,0)	15 (36,6)		

CMA: análise cromossômica por *microarrays*.

Considerando os casos de citogenética clássica reavaliados por CMA, quatro mantiveram os resultados anteriores, um caso de falha de crescimento apresentou resultado 46,XY, e um caso com resultado citogenético não foi amplificado no *array*. Considerando os resultados de CMA, seis mantiveram os resultados anteriores e uma triploidia resultou em falha de amplificação.

A aberração cromossômica mais frequentemente encontrada foi a trissomia, envolvendo os cromossomos 22 e 16 (11,1% e 4,9%, respectivamente). Não observamos anormalidades envolvendo cromossomos maiores (Grupos A e B). As frequências detalhadas das aberrações cromossômicas estão disponíveis na tabela 2. As mudanças estruturais observadas foram todas as deleções: 46,XX,del(6)(p23), 46,XX,del(12)(p13-pter) e 46,XX,del(15)(q26.2-qter)(pat). Na comparação estatística, não foi encontrada diferença significativa entre os resultados da CMA e os resultados citogenéticos, agrupados em categorias ou individualmente.

**Tabela 2:** Distribuição dos resultados obtidos por citogenética clássica e análise cromossômica por *microarrays* dos produtos da concepção

Resultados		Citogenética clássica n (%)	CMA n (%)
Normal		20 (50,0)	15 (36,6)
Inconclusivo		4 (10,0)	3 (7,3)
Trissomias			
	Trissomia 9	0	2 (4,9)
	Trissomia 13	0	1 (2,4)
	Trissomia 14	0	1 (2,4)
	Trissomia 15*	2 (5,0)	1 (2,4)
	Trissomia 16	1 (2,5)	3 (7,3)
	Trissomia 17	1 (2,5)	0
	Trissomia 18	0	1 (2,4)
	Trissomia 21	0	1 (2,4)
	Trissomia 22	2 (5,0)	7 (17,1)
	Trissomia 9 e monossomia X	0	1 (2,4)
Monossomias			
	Monosomia X*	3 (7,5)	2 (4,8)
	Monossomia 21	1 (2,5)	0
Poliploidia*		4 (10,0)	2 (4,9)
Aberração estrutural		2 (5,0)	1 (2,4)
Total		40	41

* Universal ou em apresentação em mosaico.

CMA: análise cromossômica por *microarrays*.

A idade materna no momento da perda da gravidez também foi avaliada. A tabela 3 mostra a correlação entre a idade materna e o período de perda gestacional em semanas.

**Tabela 3:** Correlação entre idade, semana de perda de gravidez e resultados encontrados pela análise citogenética clássica e análise de *microarrays* cromossômicos

Idade/perda gestacional		n (%)*	Resultados		
Até 35 anos		32 (38,3)	Normal	Anormal	Inconclusivo
	Até 5 semanas	0	0	0	0
	6-9 semanas	21 (25,9)	9	11	1
	10-12 semanas	5 (6,2)	4	1	0
	13-19 semanas	2 (2,4)	2	0	0
	Mais de 20 semanas	0	0	0	0
	Sem informação IG	4 (4,9)	1	2	1
Mais de 35 anos		36 (44,4)	Normal	Anormal	Inconclusivo
	Até 5 semanas	1 (1,2)	1	0	0
	6-9 semanas	27 (33,3)	7	17	3
	10-12 semanas	1 (1,2)	0	1	0
	13-19 semanas	1 (1,2)	0	1	0
	Mais de 20 semanas	1 (1,2)	0	0	1
	Sem informação IG	5 (6,2)	3	2	0
Sem informações de idade materna		13 (16)	Normal	Anormal	Inconclusivo
	Até 5 semanas	0	0	0	0
	6-9 semanas	5 (6,2)	3	1	1
	10-12 semanas	1 (1,2)	1	0	0
	13-19 semanas	0	0	0	0
	Mais de 20 semanas	2 (2,4)	1	1	0
	Sem informação IG	5 (6,2)	4	1	0
Total		81 (100)	36	38	7

* Em comparação com o total.

IG: idade gestacional.

Um gráfico foi construído para auxiliar na tomada de decisão de médicos e pacientes (Figura 1).

**Figura 1:** Quadro de tomada de decisão considerando a análise do produto da concepção

## DISCUSSÃO

O aborto espontâneo é a principal complicação da gravidez e um evento estressante para o casal. Saber a causa do aborto espontâneo auxilia no processo de restabelecimento do casal, afastando eventuais culpas e permitindo que planejem seu futuro reprodutivo.

Já se sabe que de 45% a 70% dos abortos espontâneos precoces são decorrentes de anormalidades cromossômicas no embrião decorrentes de eventos maternos não disjuncionais.^(^^9^^-^^11^^)^ Devido ao seu grande impacto na formação fetal, a frequência de aberrações cromossômicas diminui durante a gravidez.^(^^12^^)^ Em nossa amostra, observamos que a maioria dos abortos espontâneos ocorreu da sexta à nona semana gestacional, concentrando 66,6% das perdas gestacionais; 50,6% deles tinham aberrações cromossômicas.

A distribuição dos diversos tipos de anormalidades, levando em consideração aneuploidias, poliploidias e anormalidades estruturais, foi de 72,5%, 15% e 7,5%, respectivamente. O tipo de aneuploidia mais frequente na amostra foi a trissomia, observada em 70% dos casos anormais, 22% deles envolvendo o cromossomo 22. Uma metanálise de 19.920 casos de aborto espontâneo mostrou que a trissomia corresponde a 59% das anormalidades, a monossomia X a 15%, e a triploidia a 15%. As trissomias mais observadas por eles foram as que envolviam os cromossomos 16 (32,1%), 7 e 22 (10,7% cada).^(^^13^^)^ Além disso, uma nova pesquisa avaliou 1.920 POC e encontrou incidência de 57,2% de resultados anormais. A alteração mais frequente envolveu a trissomia do cromossomo 16, correspondendo a 12% dos casos anormais, seguida pelas trissomias dos cromossomos 21 e 22.^(^^9^^)^

Dentre as aberrações estruturais, observou-se aneuploidia parcial em um aborto tardio, ocorrido na 28ª semana de gestação. Essa variante tinha origem paterna e correspondia a uma deleção parcial do braço longo do cromossomo 15 [46,XX,del(15)(q26.2-qter)]. A deleção parcial do cromossomo 6 [(46,XX,del(6)(p23)] resultou em um aborto na sétima semana de gestação e, para a alteração do cromossomo 12 [46,XX,del(12)(p13-pter)], não temos informações sobre a idade gestacional. As três alterações foram grandes e poderiam ter sido visualizadas por ambos os métodos. A frequência de alterações estruturais é geralmente menor do que a de outras aberrações, conforme observado no presente estudo (4%) e na literatura médica. Uma revisão de 13 estudos e 7.012 casos observou frequência de 6% de rearranjos estruturais em abortos espontâneos.^(^^2^^)^

Não houve diferença no índice de sucesso entre as técnicas utilizadas: em quatro casos de citogenética, houve falha no crescimento celular, ao passo que três casos foram inconclusivos por CMA devido à amostra de DNA insuficiente para análise ou à presença exclusiva de células maternas. A falha foi observada em 8,6% da análise. O índice de falha da cultura de células para análise do cariótipo verificada neste estudo (10%) é ligeiramente diferente da taxa de 12% a 21% descrita na literatura.^(^^2^^,^^14^^)^

Treze casos selecionados aleatoriamente foram reavaliados por CMA em outro laboratório de pesquisa. Em um caso de citogenética clássica, anteriormente inconclusiva, foi possível obter resultado (46,XY). Por outro lado, um caso com resultado citogenético não foi amplificado no *array*. Talvez o material fosse insuficiente ou não tenha sido extraído corretamente. Em relação às amostras de CMA, uma triploidia resultou em falha de amplificação no segundo estudo. O nível de concordância observado foi de 66,6% e 85,7%, respectivamente.

Em relação à idade gestacional, os resultados não fornecem evidências de uma associação significativa da idade com a monossomia ou poliploidia do cromossomo sexual, mas demonstram um efeito do aumento da idade na frequência das trissomias. Hassold et al.,^(^^15^^)^ e muitos outros pesquisadores que os sucederam já tinham demonstrado a associação entre a idade materna e trissomias. Franasiak et al.,^(^^16^^)^ em um grande estudo genético de pré-implantação de embriões, observaram aumento de monossomias em pacientes com menos de 26 anos e aumento de trissomias em mulheres com mais de 34 anos.

Observamos aqui que, dentre as trissomias diagnosticadas, 73,9% ocorreram em mulheres com mais de 35 anos. A associação das trissomias com a idade materna é um problema crescente, visto que, atualmente, no Brasil a média de idade em que as mulheres têm o primeiro filho é de 31 anos, aumentando cronologicamente nas Regiões Sul e Sudeste do país (com maior acesso à educação) e se elevando proporcionalmente ao aumento do nível sociocultural.^(^^17^^)^

Em contrapartida, os resultados do abortamento em mulheres até 35 anos mostraram 48,3% com cariótipo normal, sugerindo causas de aborto diferentes das aneuploidias. Alguns estudos sugerem que o aborto sem alteração do cariótipo pode ser decorrente de outras causas (endócrinas, imunológicas, anatômicas etc).^(^^18^^)^ Mutações gênicas e mosaicismo também foram implicadas como causas das perdas gestacionais.^(^^4^^)^

Ainda considerando a idade materna, observamos que 53,7% dos abortos ocorreram em mulheres com mais de 35 anos. Entre as amostras analisadas dessas mulheres, 66,7% apresentaram resultados anormais. Vale ressaltar, entretanto, que 17% das solicitações de exames não informavam a idade da mãe, e a mediana (p.25; p.75) das idades das pacientes analisadas foi de 36 (32; 39) anos.

O cariótipo convencional ainda é considerado o padrão-ouro dos testes de POC, pois tem vantagens importantes sobre as técnicas moleculares, incluindo a detecção de tetraploidia balanceada (duas cópias da mãe e duas cópias do pai) e as translocações Robertsonianas.^(^^9^^)^ No entanto, na citogenética clássica, a análise se limita a um tempo de cultura celular que varia entre 7 e 10 dias.^(^^19^^)^ O período de análise das lâminas ainda deve ser adicionado a esse tempo, disponibilizando o resultado ao casal entre 15 dias e 1 mês, ao passo que, na análise por citogenética molecular, o resultado pode ser obtido a partir de 2 dias após o envio das amostras ao laboratório de análises, dependendo da rotina do exame. Por outro lado, no Brasil, o custo dos procedimentos é bastante diferente, e o custo da CMA é quase quatro vezes o cobrado para uma investigação citogenética clássica, além de que poucos laboratórios no país realizam tal procedimento. Esses fatores devem ser levados em consideração ao discutir com o casal o tipo de análise que deseja realizar.

Considerando as vantagens das técnicas, a CMA permite a identificação de pequenas deleções e duplicações e dissomia uniparental, mas não é capaz de detectar rearranjos cromossômicos balanceados ou poliploidia balanceada. No geral, a poliploidia é responsável por cerca de 2% a 10% de todos os abortos espontâneos,^(^^7^^)^ sendo facilmente identificável pela citogenética clássica. Em nossa amostra, foi vista em 7,4% dos casos. O procedimento de CMA permite a detecção de contaminação por DNA materno (desde que seja feita a análise do sangue materno), não necessita de cultura de células e oferece a possibilidade de avaliação de material parafinado. A exclusão da origem materna é de grande importância na análise dos resultados, sabendo-se que entre 29% e 58% dos resultados 46,XX, encontrados pela análise molecular são de origem materna,^(^^2^^,^^7^^,^^8^^,^^20^^)^ o que pode resultar em conclusões erradas sobre a análise do produto do aborto. Outra vantagem da CMA por SNPs é a possibilidade de identificar a origem parental da aneuploidia, informação que provou ser valiosa no aconselhamento reprodutivo do casal.^(^^21^^)^

Lathi et al., realizaram a análise simultânea de 30 amostras por análise citogenética clássica e por *array* SNP.^(^^21^^)^ Observaram discordância em quatro resultados (87%): o primeiro referindo-se à análise de uma amostra tetraploide e o segundo para determinar uma anormalidade derivada de translocação Robertsoniana. Em uma terceira amostra, a citogenética clássica demonstrou trissomia do cromossomo 22, e a análise de CMA identificou apenas células de origem materna. Na quarta amostra, o resultado 46,X,+22 foi observado no cariótipo, ao passo que o *array* SNP relatou 47,XX,+22.

Em uma metanálise de estudos que comparavam a CMA e a citogenética clássica, concluiu-se que houve concordância entre o *array* e a determinação do cariótipo em 86,0% dos casos. O *array* detectou 13% de anomalias cromossômicas além da citogenética clássica. Por outro lado, a citogenética detectou 3% de anormalidades adicionais em relação ao *array*.^(^^22^^)^

Sahoo et al., avaliaram 8.118 amostras de POC, tecidos frescos, fixados em formalina e embebidos em parafina usando o *array* SNP (81,6%) e o *array* de hibridização genômica comparativa (CGH) (18,4%). Foi bem-sucedida a análise de 7.396 amostras (91,1%), com 92,4% de tecido fresco e 86,4% fixados em formalina e embebidos em parafina. Foram identificadas anormalidades clinicamente significativas em 53,7% das amostras, 94% das quais consideradas como a causa da perda gestacional.^(^^23^^)^

Shah et al., analisaram 60 amostras simultaneamente por citogenética, *array* SNP e *array* de CGH. Encontraram índice geral de discordância de 33% nos resultados, devido à contaminação de células maternas, rearranjos cromossômicos balanceados, poliploidia e mosaicismo placentário (detectado em 18% de todas as amostras). Ocorreu falha de crescimento em quatro amostras enviadas para citogenética, das quais três eram cromossomicamente anormais por teste molecular.^(^^24^^)^

Independente do método escolhido, sabemos que, sem avaliação cromossômica, 80% das perdas gestacionais recorrentes permanecem sem esclarecimento etiológico. Destas, 62% teriam anormalidades cromossômicas, ao passo que os 18% restantes permaneceriam inconclusivas após o cariótipo clássico.^(^^25^^)^ Além disso, Ouyang et al., demonstraram que a chance de encontrar alterações cromossômicas no POC depende da idade gestacional, sendo mais frequente na fase embrionária do que na fetal. Também foram mais frequentes em gestações embrionárias do que em gestações não embrionárias.^(^^26^^)^

Segawa et al.,^(^^27^^)^ realizaram um importante estudo citogenético de 1.030 POC gerados após a transferência de embriões em pacientes submetidas a procedimentos de fertilização *in vitro*. Observaram que 80,6% eram aneuploides, incluindo 1% poliploide e 1,1% de cariótipos em mosaico. A poliploidia e os cariótipos normais foram significativamente mais frequentes em gestações sem batimento cardíaco fetal. Também observaram 2,4% de anormalidades estruturais, corroborando nossos resultados de aborto espontâneo.

Considerando a idade gestacional dos POC avaliados no presente estudo, 89,4% ocorreram durante o primeiro trimestre gestacional. Das oito pacientes com idade gestacional igual ou superior a 12 semanas, apenas duas (25%) apresentaram alterações genéticas (uma detectada pela citogenética e outra detectada pela CMA), e uma não apresentou crescimento celular.

Reconhecemos como limitações de nosso estudo o fato de que poucas amostras foram submetidas a ambos os testes. Embora não possamos comparar os resultados diretamente, não observamos frequência diferente de aneuploidias entre os grupos ou resultados que seriam observados por apenas um método. Mesmo os rearranjos estruturais poderiam ter sido detectados por ambas as metodologias. Além disso, apenas a citogenética clássica indicou a presença de mosaicismo, mas as técnicas moleculares atuais permitem essa detecção, desde que em proporção maior que 15%.

Assim, considerando os dados citados, sugere-se que os POC sejam avaliados primeiramente pela citogenética clássica, considerada padrão-ouro na análise de POC, preservando (tecidos congelados ou fixados em formalina e embebidos em parafina), quando possível, parte do material genético para posterior análise molecular nos casos com resultados 46,XX, resultados normais com clínica incompatível ou com falha de cultura celular, principalmente em mulheres com mais de 35 anos.

## CONCLUSÃO

Não houve diferença nos resultados da análise cromossômica por *microarrays* e da citogenética clássica quanto à frequência das aberrações cromossômicas observadas. Foram encontrados resultados inconclusivos em proporção semelhante em ambas as técnicas. Foram observados resultados anormais com maior frequência até a nona semana de gestação e em mulheres com 35 anos ou mais.
